# Okra [*Abelmoschus esculentus* (L.) Moench] improved blood glucose and restored histopathological alterations in splenic tissues in a rat model with streptozotocin-induced type 1 diabetes through CD8^+^ T cells and NF-kβ expression

**DOI:** 10.3389/fvets.2023.1268968

**Published:** 2023-11-16

**Authors:** Mohamed A. Alblihd, Khalaf F. Alsharif, Asmaa A. Hamad, Fatma Abo Zakaib Ali, Manal T. Hussein, Alaa S. Alhegaili, Mohamed Ahmed Hassan, Osama M. Al-Amer, Nisreen Khalid Aref Albezrah, Abdulraheem Ali Almalki, Alaa Jameel A. Albarakati, Khalid S. Alghamdi, Khalid J. Alzahrani, Ashraf Albrakati, Elham Hamed Alrubai, Naira ElAshmouny, Ehab Kotb Elmahallawy

**Affiliations:** ^1^Department of Medical Microbiology and Immunology, College of Medicine, Taif University, Taif, Saudi Arabia; ^2^High Altitude Research Center, Taif University, Taif, Saudi Arabia; ^3^Department of Clinical Laboratories Sciences, College of Applied Medical Sciences, Taif University, Taif, Saudi Arabia; ^4^Department of Biology, College of Science, Taif University, Taif, Saudi Arabia; ^5^Department of Pathology and Clinical Pathology, Faculty of Veterinary Medicine, Sohag University, Sohag, Egypt; ^6^Department of Cell and Tissues, Faculty of Veterinary Medicine, Assiut University, Asyut, Egypt; ^7^Department of Medical Laboratory, College of Applied Medical Sciences, Prince Sattam bin Abdulaziz University, Alkharj, Saudi Arabia; ^8^Food Science and Technology Department, Faculty of Agriculture, Al-Azhar University – Assiut Branch, Asyut, Egypt; ^9^Department of Medical Laboratory Technology, Faculty of Applied Medical Sciences, University of Tabuk, Tabuk, Saudi Arabia; ^10^Genome and Biotechnology Unit, Faculty of Sciences, University of Tabuk, Tabuk, Saudi Arabia; ^11^Department of Obstetrics and Gynecology, College of Medicine, Taif University, Taif, Saudi Arabia; ^12^Department of Clinical Laboratory Sciences, College of Applied Medical Sciences, Taif University, Taif, Saudi Arabia; ^13^Surgery Department, College of Medicine, Al-Qunfudah Branch, Umm Al-Qura University, Makkah, Saudi Arabia; ^14^Forensic Poison Services Administration, Forensic Medical Services Center in Taif, Ministry of Health Saudi Arabia, Taif, Saudi Arabia; ^15^Department of Clinical Laboratory Sciences, College of Applied Medical Sciences, Taif University, Taif, Saudi Arabia; ^16^Department of Human Anatomy, College of Medicine, Taif University, Taif, Saudi Arabia; ^17^Internal Medicine Department, Security Forces Hospital, Riyadh, Saudi Arabia; ^18^Department of Histology and Cell Biology, Faculty of Medicine, Kafrelsheikh University, Kafr El Sheikh, Egypt; ^19^Departamento de Sanidad Animal, Grupo de Investigación en Sanidad Animal y Zoonosis (GISAZ), Facultad de Veterinaria, Universidad de Córdoba, Córdoba, Spain; ^20^Department of Zoonoses, Faculty of Veterinary Medicine, Sohag University, Sohag, Egypt

**Keywords:** diabetes, okra, white pulp, lymphocyte, hemosiderosis, NF-kβ

## Abstract

Diabetes mellitus is a complex metabolic syndrome that involves dysfunction of spleen and other lymphoid organs. Medicinal plants, including okra (*Abelmoschus esculentus* (L.) Moench), were used widely for diabetes treatment. Scarce data are available about the potential anti-diabetic effects of okra, the histopathological alterations in splenic tissues and the mechanistic pathways underlying this association. The current research investigated the effects of okra pod extract on the biochemical parameters and expression of CD8^+^ T cells and nuclear factor kappa (NF-k) B and releasing proinflammatory cytokines in spleen in streptozotocin (STZ)-induced diabetic rat models. A total of 50 mature male Wister albino rats were divided into five isolated groups; the first served as control (untreated) animals, the second (DM group) diabetes induced by STZ (at a dose of 45 mg/kg body weight, administered intraperitoneally), the third group (DM + Insulin): diabetic rats administered insulin subcutaneously (10 units/kg bw/day) daily for 4 weeks, the fourth group was administrated 400 mg/kg okra extract daily for 4 weeks, and diabetic induced rats in the fifth group were administrated 400 mg/kg okra extract daily for 4 weeks. The 2,2-diphenyl-1-picrylhydrazyl (DPPH) scavenging activity in *Abelmoschus esculentus* (L.) Moench was studied, and the content of phenolic compounds in okra pods was estimated using high-performance liquid chromatography. Diabetes induction led to decreased body weight, increased blood glucose levels. Capsular thickness was significantly increased, white pulp was widely dispersed, and mature lymphocytes in the periphery were also drastically decreased, with thick follicular arteries, necrosis, and depletion of lymphocytes in the germinal center. Red pulp revealed severe congestion and degenerative changes, deposition of hemosiderin granules and lymphocytic depletion. In addition, collagen fiber deposition was increased also in this group. The induction of diabetes exaggerated NF-kβ expression and mediated downregulation of the expression of CD8^+^ T cells in spleen tissue. Interestingly, oral administration of okra extracts post diabetes induction could mitigate and reverse such adverse effects. Altogether, our study points out the potential benefits of okra in improving blood glucose levels and restoring histopathological alterations in splenic tissues through CD8^+^ T cells and NF-kβ expression in a diabetic rat model.

## 1 Introduction

Diabetes is a significant non-communicable illness with various etiologies (1). The World Health Organization (WHO) estimated that diabetes mellitus (DM) affected more than 500 million people worldwide ages 20–79 years in 2021 (1). Uncontrolled diabetes can negatively affect many body systems, including the nervous and vascular systems, resulting in catastrophic complications. It is one of the top five killers worldwide, and it can cause blindness, kidney failure, and heart attack and lead to lower limb amputation (2, 3). Type 1 diabetes (T1D) is characterized by multiple imperfections in humoral and cellular immunity (4, 5). Type 1 diabetes mellitus (T1DM), also known as insulin-dependent diabetes mellitus (IDDM), is claimed to affect about 10% of clinically diagnosed diabetic patients (6). According to previous studies (7), viruses, chemical pollutants, and autoimmune reactions can cause pancreatic β cells to produce insufficient amounts of insulin, which leads to T1DM. Uncontrolled hyperglycemia disrupts organ structure and function (8, 9). Additionally, hyperglycemia has been considered a critical mediator of altered lymphocyte function, which might drive the induction of oxidative stress besides play a key role in impairment of immunological responses following diabetes (10, 11). Diabetes is a complicated metabolic disorder marked by dysfunction of the immune system as well as failure of the lymphoid organs, especially the spleen (12). Increased production of reactive oxygen species (ROS) can aggravate inflammation by activating NF-kB. leading to a rise in proinflammatory cytokine levels and, as a result, cellular damage (13). Furthermore, diabetes causes thymus atrophy due to lymphocyte depletion (14). Based on their safety and lack of harmful side effects, the use of medicinal plants or natural substances for self-medication has expanded significantly, especially in developing or low-income nations (15–17). Recent research has focused on flavonoids derived from natural sources, which have no toxicity or adverse effects and represent a comparatively less expensive novel approach to slow the progression of diabetes (18). Okra (*Abelmoschus esculentus*), also known as lady fingers, or bamia in Egypt, is a common vegetable plant growing in tropical and subtropical regions of the world (19). It has recently spread throughout the world, but its planting and consumption are more common in Egypt, China, Cyprus, Greece, and Turkey (20, 21). Okra provides basic nutrients such as vitamins, minerals, dietary fiber, and dietary supplements. The peel and seeds have been documented to have anti-diabetic and anti-hyperlipidemic effects in streptozotocin-induced diabetic rats (22). According to Deters et al. (23), okra can lower blood glucose and cholesterol levels in obese mice. In addition, it is involved in hepatoprotection and ulcer healing, and has anti-cancer, anti-inflammatory, and laxative functions (24). Okra’s anti-diabetic properties are attributable to flavonoids in the plant, such as quercetin, which have antioxidant properties and protect cells from oxidative stress. As a result, it can both repair injured beta cells and reduce the total number of cells (25). Okra can also boost insulin secretion and ameliorate insulin resistance (26).

When flavonoids are taken orally, they are most effective in treating the pancreas and its beta cells, and, in turn, diabetes (25). However, the underlying mechanism of the regulating potential of phenolic compounds in okra with regard to their anti-hyperglycemic activity remains unknown. Glucosamine-nitrosourea chemical compound, called streptozotocin (STZ), derived from the bacterium *Streptomyces achromogenes* has been used to treat pancreatic cancer besides various chemotherapeutic purposes. Pancreatic cells are destroyed by STZ, which induces polydipsia, polyuria, hypoinsulinemia, and hyperglycemia, producing type 1 diabetes mellitus (4, 27). Against this background, our study was intended to investigate the anti-diabetic effects of phenolic compounds in okra pod (OP) extract on rats with STZ-induced T1DM and examine the effect of diabetes and the course of treatment on their splenic immune system. In addition, we aimed to examine the effect of okra in STZ-induced diabetic animals using parameters including body weight, fasting blood glucose level, glycosylated hemoglobin, and spleen tissue histopathological examination. Moreover, the potential clinical implications of CD8 and NF-kB were also evaluated.

## 2 Materials and methods

### 2.1 Materials

#### 2.1.1 Materials and reagents

Ethanol, gallic acid, quercetin, 1,1-diphenyl-2-picrylhydrazyl (DPPH) radical, Folin–Ciocalteu reagent, sodium nitrite, aluminum chloride, sodium hydroxide, and sodium bicarbonate were of analytic grade and provided by the Faculty of Science, Alazhar University (Assiut Branch), Egypt.

#### 2.1.2 Preparation of okra pods extract

Small fresh green okra pods (*Abelmoschus esculentus*) were purchased from a local market (Sohag City, Sohag, Egypt). The inedible sections of the okra pods were removed before washing them with clean tap water. They were dried in the shade in a thin layer at room temperature, then ground with a laboratory mill (Braun, Germany). A total of 100 g dried powder from okra pods was immersed in 1,000 mL of 70% ethanol and stirred (by a Mettler magnetic stirrer) for 3 h at room temperature. The extracts were centrifuged for 10 min at 5,000 rpm after being extracted twice more as above. Following the extraction, all supernatants were collected and condensed to dry residue using a rotary evaporator under vacuum at 40°C. The concentrated samples were lyophilized (dried at 45°C under negative vacuum) to obtain okra pod powder, which was stored at 20°C until analysis.

#### 2.1.3 Quantitative identification of phenolic compounds in okra pods and high-performance liquid chromatography analysis

This step involved identifying the phenolic compounds in okra pod extract using high-performance liquid chromatography (HPLC) as described elsewhere (28). This phase was carried out using a double piston pump (Beckman model 126) and a fluorescence detector (Perkin Elmer LC 240); a reaction pump (Dioxin); a derivatization tube 10 × 0.33 mm; a data processing system (Gold Data Management); a SUPELCOSIL LC-18-DB column, 25 cm × 4.6 mm × 5 m; and a 20 L injector (Beckman). The UV detector was set to 272 nm, and each compound was identified by comparing retention times and UV/VIS spectra to standards. The calibration curves of the respective standards were used to quantify the compounds.

#### 2.1.4 Determination of total phenolic compound content

The Folin–Ciocalteu method was used to calculate the total phenolic component contents (29). The Folin–Ciocalteu reagent was diluted with deionized water (1:10), and 0.750 mL of sodium bicarbonate solution (7.5% w/v) were added to a 0.1 mL sample (1.5 mg/mL). The mixture was incubated for 90 min at room temperature (dark conditions). The combination’s absorbance was measured at 765 nm using a UV–visible spectrophotometer Then phenolic contents were expressed as grams of gallic acid equivalents (GAE) per gram of extract.

#### 2.1.5 Determination of total flavonoid content

The Dewanto et al. (30) technique was used to assess the total flavonoid content. This step involved mixing of 2.25 mL of distilled water, 0.15 mL of 5% NaNO2 solution, and 0.5 mL of sample extract in a test tube. Then, after 5 min, 0.3 mL of AlCl_3_6H_2_O solution (10%) was added after vertexing for 6 min. The addition of 1.0 mL of 1 M NaOH was done followed by completely mixing with a vortex. The absorbance was then measured right away at 510 nm. Quercetin equivalents (QE) per gram of dry material (mg/g) were used to represent the results.

#### 2.1.6 DPPH radical scavenging assay

The Pothitirat et al. (31) approach was used to conduct the DPPH radical scavenging test. In this step, 2 mL of the samples were diluted in different concentrations of the extraction solvent (0.25–1.5 mg/mL) and then mixed with 2 mL of DPPH solution (0.1 mM, in ethanol). Following vortex for 30 min at room temperature, the reaction mixture was incubated in the darkness, and the absorbance was determined at 517 nm in comparison to a control. The preparation of the control group was identical to that of the test group, with the exception that the antioxidant solution was replaced with an equivalent extraction solvent. The following formula was used to determine how much the sample inhibited the DPPH radical:


$$\begin{array}{l} \mathrm{DPPH}\;\mathrm{scavenging}\;\mathrm{activity}\;\left(\%\right)\\ =\left[\left(\mathrm{Ab}\;\mathrm{control}-\mathrm{Ab}\;\mathrm{sample}\right)/\mathrm{Ab}\;\mathrm{control}\right]\;\times \;100 \end{array}$$

#### 2.1.7 Drugs and chemicals

Sigma-Aldrich Company (St. Louis, MO, USA) provided the streptozotocin powder, trisodium citrate dihydrate, and citric acid monohydrate.

### 2.2 Animals

Fifty adults male Wister rats, weighing between 165 and 200 g, were obtained from the Experimental Animal House of Sohag University. Rats used in this study were kept in clean stainless-steel cages with a 12 h light/12 h dark cycle, five rats to a cage. Throughout the experiment, they were given a conventional pellet meal and unlimited amounts of water. To maintain a clean environment, bedding was changed on a regular basis. The rats were given a week for acclimatization before to the experiment’s start.

### 2.3 Methods

#### 2.3.1 Ethical considerations

Research protocols were carried out in accordance with the Declaration of Helsinki and Taif University’s ethical standards, which were both approved by Taif university’s ethics committee, Taif, Saudi Arabia (approval number HAO-02-T-105).

#### 2.3.2 Experimental design

Rats were monitored for 1 week prior to the experiment to prevent the inclusion of parasitically infected animals. During this week, samples of each group’s feces were evaluated using concentration floatation and sedimentation concentration techniques to exclude animals infested by parasites. Following acclimatization, rats were randomly assigned to 4 different isolated groups (10 animals per each) as follows:

➢ Group 1: Normal control (Control; *n* = 10): fed standard rat chow and drinking water.
➢ Group 2: DM (*n* = 10): fasted overnight (12 h before induction of diabetes).
➢ Group 3: DM + Insulin (*n* = 10): diabetes induced (same as diabetic positive control group 2), insulin administered subcutaneously (10 units/kg bw/day) (32) daily for 4 weeks.
➢ Group 4: Okra (*n* = 10): 400 mg/kg okra extract administered daily for 4 weeks by gavage (18, 33).
➢ Group 5: DM + Okra (*n* = 10): 400 mg/kg okra extract administered daily for 4 weeks by gavage (18, 33); same treatment as group 4 after STZ treatment (same as diabetic positive control group 2). By the end of the experimental protocol and after 12 h of the final treatment with extract, all rats were sacrificed and blood and tissue samples were collected.

#### 2.3.3 Induction of diabetes

As previously described (34, 35), diabetes was established by intraperitoneal injection of 45 mg/kg of streptozotocin (0.1 M cold citrate buffer, pH 4.5). The rats were allowed free access to food and water after the injection and were instructed to drink a 15% glucose solution all night long to prevent hypoglycemia. Within 3–6 days after STZ treatment, blood glucose levels were assessed (36–39). Diabetes was diagnosed by polydipsia, polyuria, and blood glucose levels 72 h after STZ injection using blood samples collected by tail prick Using tail prick blood samples and a glucometer (On Call Plus, ACON Laboratories, Germany),diabetes was diagnosed by observation of polydipsia, polyuria, and high blood glucose levels 72 h after STZ injection. The term “diabetic” only refers to STZ-injected rats with blood glucose levels of 250 mg/dL or higher. During the course of the trial, we observed various diabetic symptoms such as polydipsia, substantially elevated polyuria, and frequent urination in addition to elevated blood glucose levels. Following diabetes confirmation, rats with hyperglycemia (blood glucose >250 mg/dL) were collected for the study.

#### 2.3.4 Measurement of animal body weight

The initial body weights of all experimental animals were recorded, and the final body weights were recorded at the time of sacrifice.

#### 2.3.5 Sample collection

##### 2.3.5.1 Evaluation of fasting blood sugar

Fasting blood glucose levels were measured during the experiment using a modified version of the method described in previous work (40), which involved drawing blood via a tail prick following sanitizing the area with 10% alcohol, and allowing the blood to touch the test strip. The test strip was then placed into a calibrated glucose meter (On Call Plus Glucometer, ACON Laboratories, Germany). After 5 s, a direct reading in mg/dL was provided.

##### 2.3.5.2 Whole blood samples

At the completion of the experiment, rats were euthanized and sacrificed individual blood samples from each group were taken in dry clean tubes containing EDTA (anticoagulant) for measuring glycosylated hemoglobin (HbA1C) (41).

#### 2.3.6 Biochemical analysis

##### 2.3.6.1 Fasting blood glucose level measurement

In this step, the fasting blood glucose levels were continuously checked during the trial (40).

##### 2.3.6.2 Cumulative blood sugar measurement

The ARKRAY ADAMS A1c HA-8190 V, based on high-performance liquid chromatography (hHPLC), is a fully automated hemoglobin (HbA1c) analyzer. Automated detection and separation of variable hemoglobin is performed by the HA-8190 V (42).

##### 2.3.6.3 Histopathological examination

Following the completion of the experiment, the animals were euthanized, and tissue samples, principally spleen, were taken, dissected, and quickly fixed in 10% formalin for 24 h, dehydrated in a graded alcohol series, cleaned in xylene, and embedded in paraffin. Hematoxylin and eosin (H&E) were used to stain tissue sections, which were cut into 3 μm thick sections (43). For histological analysis, the collagen and iron deposits in the splenic tissue were identified using Masson’s trichrome and Perl’s Prussian staining, respectively. All sections were evaluated with an Olympus light microscope (Olympus CX43 light microscope) and taken using a camera (Olympus SC52) adapted for the microscope.

##### 2.3.6.4 Morphometric study

A total of 10 slides from each group were examined under low-power objective and chosen to measure the thickness of the splenic capsule using ImageJ software (34, 35). Organ histology analysis was performed, and scores were assigned based on the severity of damage seen in the analyzed tissue in each group, as previously established: 0 = no lesions; 1 = mild (1 to 25%); 2 = moderate (26 to 45%); and 3 = severe (>45%) (44, 45).

A simple approach for quantifying collagen fibers in atherosclerotic lesions is to use Masson’s trichrome and Perl’s Prussian blue staining. This method is based on open-source ImageJ software and the color deconvolution plugin. The original images of lesions were transformed to RGB images, which were then deconvolved by ImageJ using the color deconvolution plugin. The resulting monochromatic images showed collagen fibers (Masson’s trichrome) and/or iron overload (Perl’s Prussian blue staining) at maximum separation from background tissues. Collagen fibers were accurately and efficiently quantified in order to quantify the area of the green component. We measured the area of interest after selecting “Image” from the menu, choosing the “Adjust” box, and using the “Threshold” tool to isolate the green collagen fibers or the blue iron area. Depending on the stain, the threshold was manually adjusted until the entire green or blue region was highlighted in red. The threshold area was then measured by entering the set measurement dialogue under the “Analyze” menu, and after checking “Area,” “Integrated Intensity,” and “Limit to Threshold,” clicking the “Measurement” button under the “Analyze” menu, and the results were displayed in the “Results” window. Finally, morphometry was performed and area-based percentage analysis was used (46).

##### 2.3.6.5 Immunohistochemistry

Immunohistochemistry was performed as described by Attaai et al. (47). Mouse polyclonal anti-CD8 (1:200; Abcam, catalog no. ab4055) and rabbit monoclonal anti-NF-kβ (1:100; Cell Signaling, catalog no. 8242) was used as a primary antibody. Sections were incubated with the secondary antibody, Ultra Tek HRP Anti-Polyvalent Staining System (goat anti-mouse, rat, rabbit, and guinea pig IgG), which was purchased from ScyTek (USA), followed by incubation with VECTASTAIN ABC (avidin–biotin complex) reagent in a humid chamber at room temperature for 45 min. The reaction was visualized using 0.04% 3,3′-diaminobenzidine and 0.003% H_2_O_2_ in Tris–HCl buffer (0.05 M; pH 7.5) for 5–10 min. Harris hematoxylin was used as a counterstain on the sections for 30 s. Following sections dehydration in ascending concentrations of ethanol, they were cleaned in xylene and covered using DPX mounting media. Leitz Dialux 20 microscope was used to analyze the immunohistochemical staining, and a Canon Powershot A95 digital camera was used to capture pictures.

A minimum of three fields from a minimum of three different rats were quantified. For each rat, 15 randomly chosen splenic portions were used for the measurements. NF-k and CD8 expression were analyzed quantitatively using Image J (Version 1.53i) program. Image J software was used to quantify the DAB signal in order to calculate the variations in immunoreactivity. Select “Set Measurement” from the “Analyze menu,” then check “Area,” “Max. gray value,” and “Mean gray value” in the pop-up box that appears. In the cytoplasm of the cells, many circles were drawn and recorded. The findings were copied into an excel sheet, and the optical density was determined using the formula shown below: To determine how dark the stained cells are in response to the DAB signal, use the formula OD = log (Max. gray intensity/mean gray intensity) (48).

### 2.4 Statistical analysis

The measurements from the experimental groups were statistically estimated using GraphPad Prism, version 5 (San Diego, CA, USA) with one-way ANOVA and with Tukey’s *post hoc* multiple comparison tests; P 0.05 was used in the data to define statistical significance between groups (54, 55). The data were expressed as mean with standard deviation (SD), and the measurements obtained from the experimental groups were expressed as mean with SD.

## 3 Results

### 3.1 Identification of DPPH scavenging activity and phenolic compound content of okra pods using HPLC

The DPPH scavenging activity of okra pods is illustrated in Table 1. Table 2 shows the potential bioactive phenolic compounds of okra pods using HPLC. As shown in the table, eight compounds were identified: catechin, chlorogenic acid, p-coumaric acid, ferulic acid, gallic acid, caffeic acid, quercetin and kaempferol.

**Table 1:** The DPPH scavenging activity of okra pods.

**Samples**	**Total phenolic content (mg GAE/g)**	**Total flavonoid content (mg QE /g)**	**IC**_**50**_ **of DPPH radical (mg/ml)**
Okra pods	46.45	11.61	0.83

**Table 2:** Phenolic compounds contents in okra pods extract using HPLC.

**Component**	**Concentration (mg/g)**	**%**
Catechin	45.69	4.57
Chlorogenic acid	27.45	2.75
p-Coumaric acid	17.82	1.78
Ferulic acid	7.50	0.75
Gallic acid	4.22	0.42
Caffeic acid	1.15	0.12
Quercetin	1.77	0.18
Kaempferol	0.02	0.015

### 3.2 Diabetic induction and body weight measurement

**Figure 1:**
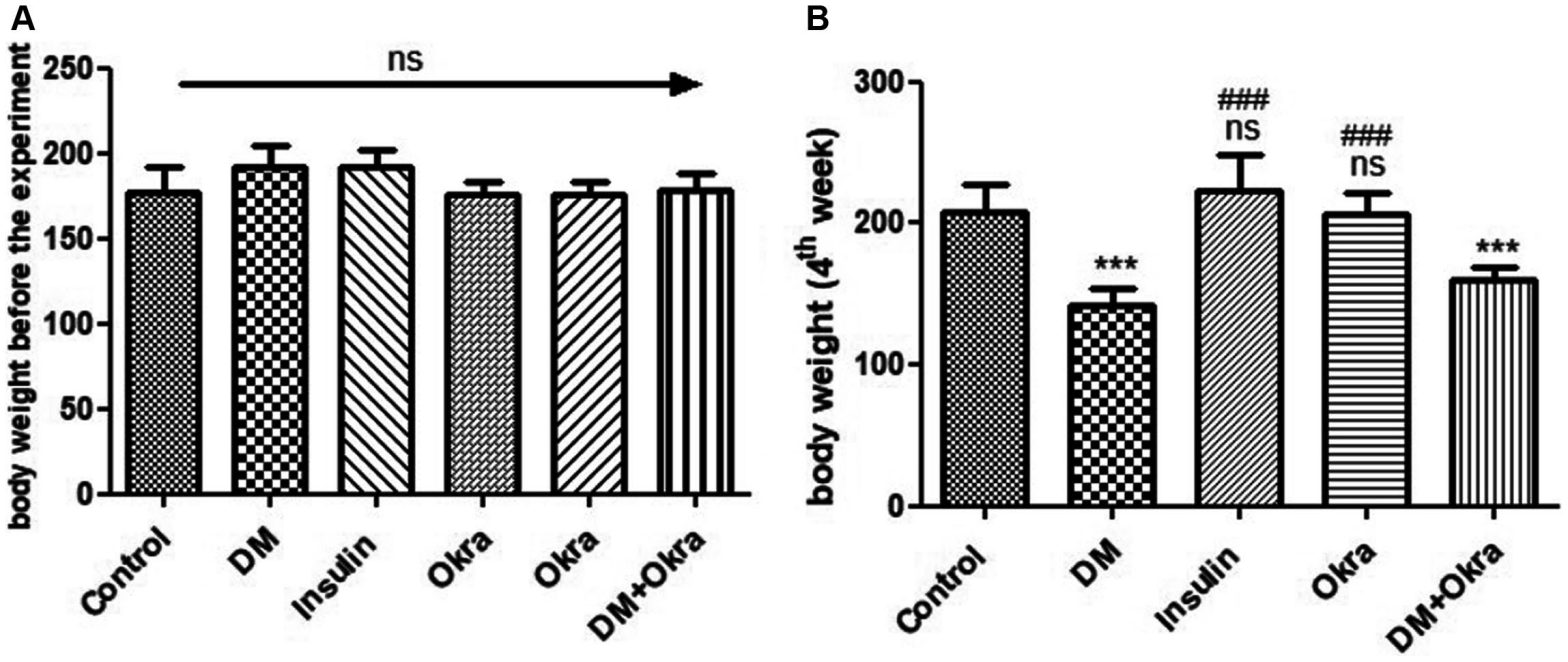
Comparisons in the body weights between experimental groups in STZ DM model: **(A)**: Body weight before the experiment, **(B)**: Body weight (4th week). Values are expressed in Means ± SD. Significant differences vs. the control group are marked by different asterisks, while significant differences versus DM group are marked by different # through one-way ANOVA with Tukey’s *post hoc* test: ^###,^ *** *p* ≤ 0.001; ns: non-significant vs. control.

Experimental Animals developed type 1 diabetes within 72 h, and displayed typical DM symptoms like polyphagia, polydipsia, and polyuria as well as significant (P 0.05) unexplained weight loss that began at the end of the first week and continued until the end of the experimental period before sacrifice (Figure 1). The significance (*p* ≤ 0.05) of decreased body weight was much more obvious in the untreated diabetic group than in the DM + Okra treated group, compared with the control group. on the other side, the diabetic rats treated with insulin showed normal body weight at the end of the experiment (Figure 1B).

### 3.3 Biochemical assessment

**Figure 2:**
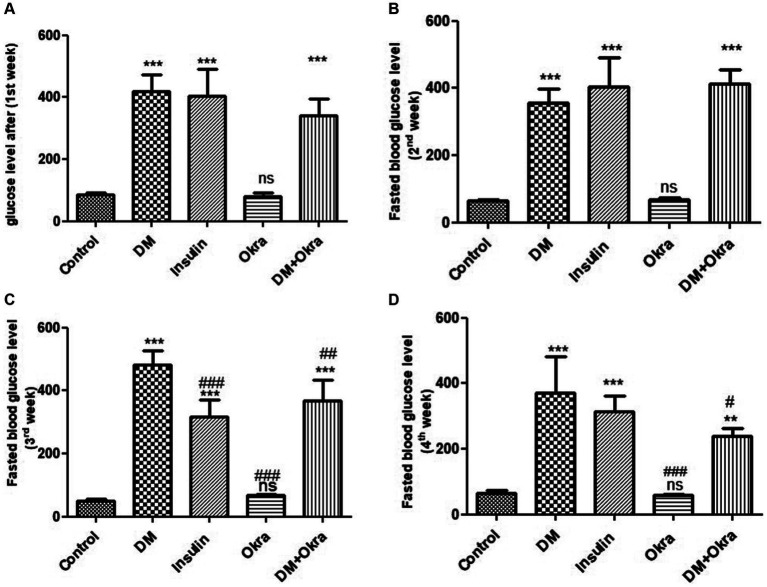
Comparisons in the fasting blood glucose level of between experimental groups in STZ DM model. **(A)**: glucose level (1st week), **(B)**: Fasted blood glucose level (2nd week), **(C)**: Fasted blood glucose level (3rd week), **(D)**: Fasted blood glucose level (4th week). Values are expressed in Means ± SD. Significant differences vs. the control group are marked by different asterisks,while significant differences versus DM group are marked by different # through one-way ANOVA with Tukey’s *post hoc* test: ^#^^,^ *p ≤ 0.05, ^##^^,^ **p ≤ 0.01, ^###^^,^ ***p ≤ 0.001, ns: non-significant vs. control.

**Figure 3:**
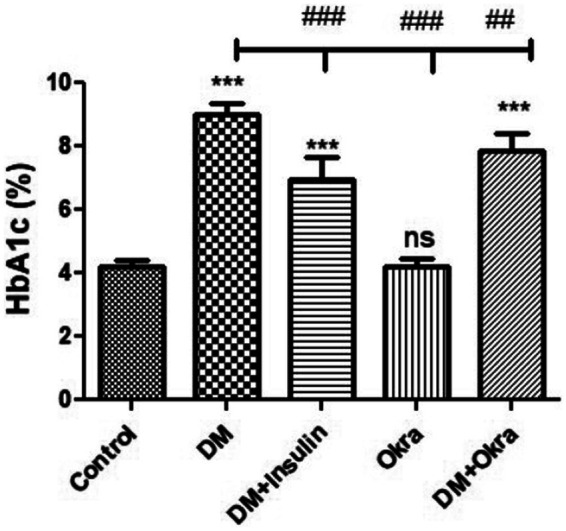
Comparisons in the cumulative blood sugar value (HbA1C) of between experimental groups in STZ DM model: Values are expressed in Means ± SD. Significant differences vs. the control group are marked by different asterisks,while significant differences versus DM group are marked by different # through one-way ANOVA with Tukey’s *post hoc* test: ^##^*p* ≤ 0.01, ^###,^ ***p ≤ 0.001, ns: non-significant vs. control.

Statistical analysis of fasting blood sugar during the first, second, and third weeks of the experiment revealed that both diabetic (DM) groups, those treated with okra and those treated with insulin, had significantly (*p* ≤ 0.05) increased levels compared to the negative control and okra control groups (Figure 2). At the end of the fourth week and before sacrifice, the mean value for the DM + Okra treated group was significantly (*p* ≤ 0.05) decreased compared to untreated DM group, and approached the level of the negative control and okra control groups; however, the elevated blood sugar levels in this group were still significant compared with the control groups (Figure 2). The highest cumulative blood sugar (HbA1c) level was observed in the DM group, which was significantly (*p* ≤ 0.05) higher compared to the negative control and okra control groups; it also reflected the diabetic status of rats in this group. The means cumulative blood sugar level was significantly (*p* ≤ 0.05) higher in the DM + Okra treated group compared to the negative control and okra control groups, and significantly lower compared to the DM group (Figure 3).

### 3.4 Histopathological assessment

**Figure 4:**
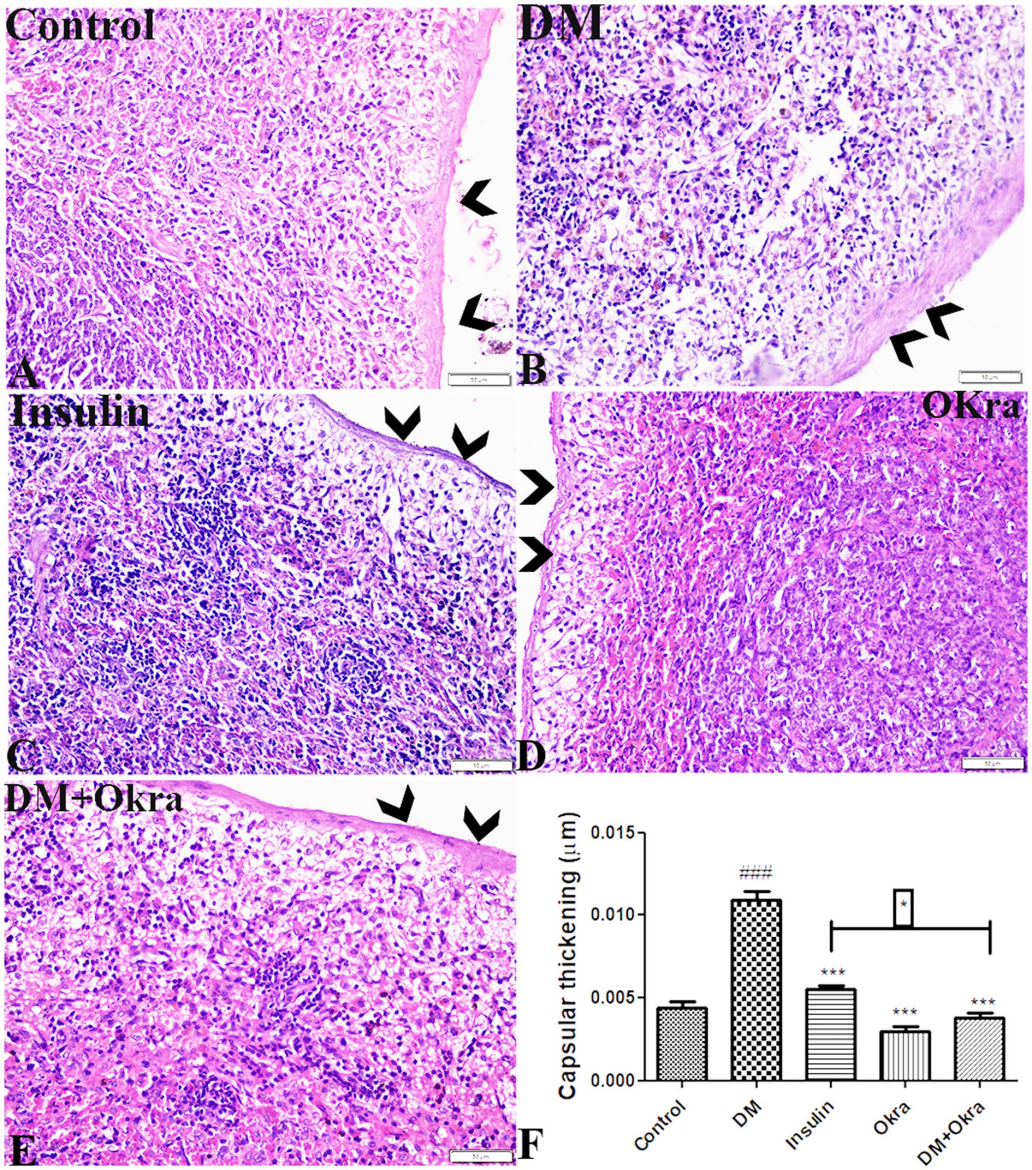
Photomicrograph of spleen tissue sections stained with HE stains from **(A)** control group, **(B)** DM untreated group, **(C)** diabetic group treated with insulin, **(D)** okra control group, **(E)** DM + Okra, showing the capsular thickness. The bar size represents 50 μm. **(F)** Histomorphometry graph showing quantitative measurements of splenic capsular thickness. Data are expressed as means ± standard deviations. Significant differences versus the control group are marked by #, while significant differences versus DM group are marked by different asterisks through one-way ANOVA with Tukey’s *post hoc* test: *p ≤ 0.05, ^###,^ ***p ≤ 0.001.

Microscopic analysis of spleen sections stained with H&E revealed normal histological appearance of the splenic capsular structure and thickness in the control group (G1) and the okra control group (G4) (Figures 4A,D). Capsular thickness was significantly increased in untreated DM animals compared with the control groups (Figures 4B,F). A significant reduction in spleen capsular thickness was observed in diabetic animals treated with insulin (Figure 4C). However, the restoration and improvement in capsular thickness was more obvious in the DM + Okra group (Figures 4E,F).

**Figure 5:**
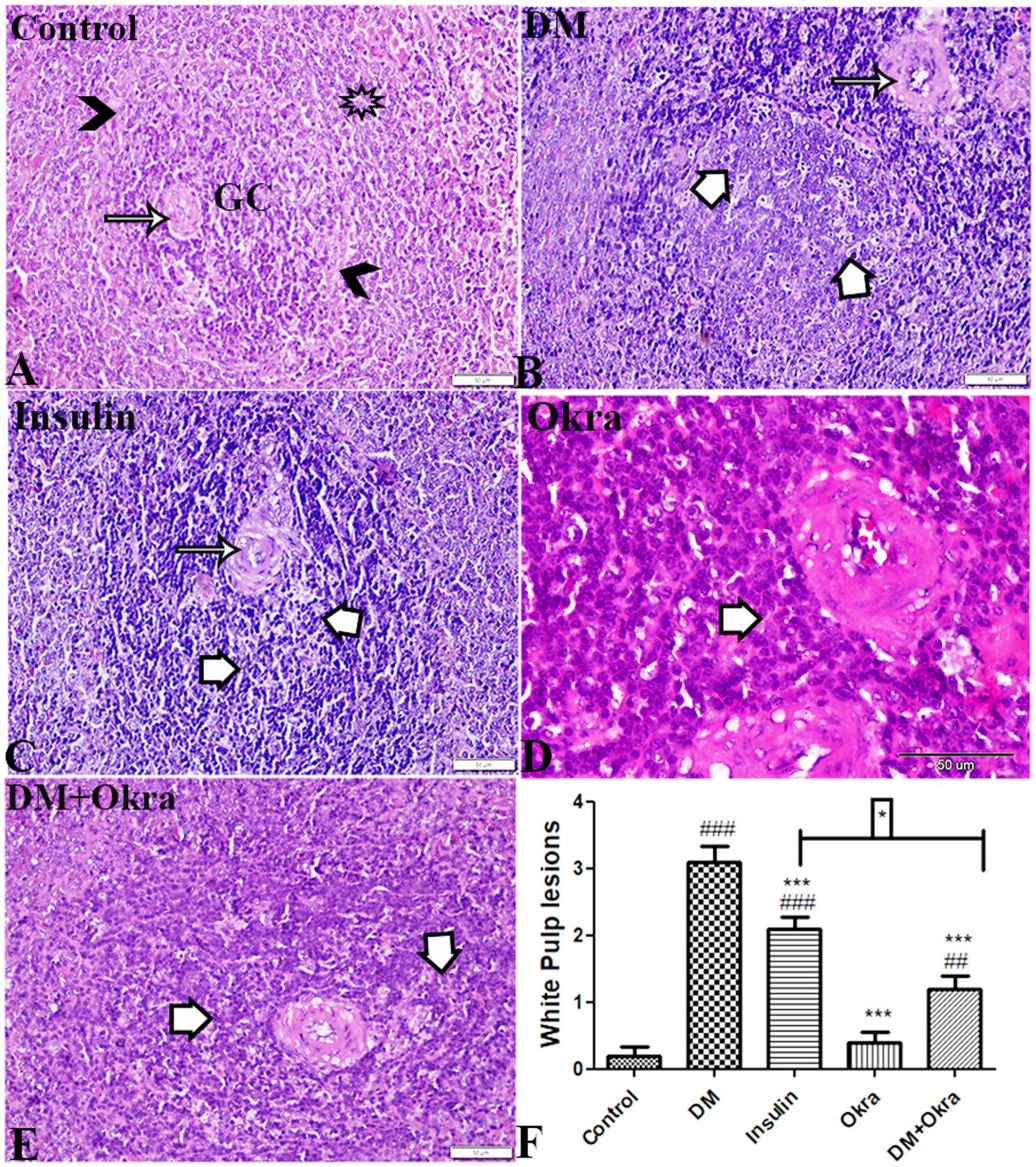
Photomicrograph of spleen tissue sections (White Pulp) stained with HE stains **(A)** control group: normal size and structured white pulp, normal follicular artery (arrow), germinal center lymphocytic cellular density (GC), Mantle zone (arrowheads) surrounded by marginal zone (star). **(B)** DM untreated group: thick follicular artery (arrow), necrosis and depletion in lymphocytes of germinal center (white arrows). **(C)** Diabetic group treated with insulin: mild thickness in follicular artery (arrow), lymphocytic depletion in germinal center (white arrows). **(D)** Okra control group: normal white pulp structure and cellular density. **(E)**: DM + Okra, showing improvement in white pulp structure, mild lymphocytic cellular depletion in germinal center (white arrows). The bar size represents 50 μm. **(F)** Histomorphometry graph showing semiquantitative measurements of splenic white pulp total lesion scores. Data are expressed as means ± standard deviations. Significant differences versus the control group are marked by #, while significant differences versus DM group are marked by different asterisks through one-way ANOVA with Tukey’s *post hoc* test: *p ≤ 0.05, ^###,^ ***p ≤ 0.001.

The histological structure of white pulp is composed of follicular arteries with condensed lymphocytes and periarterial lymphatic sheath (PALS), and the germinal center contains condensed lymphocytes and a mantle zone surrounded by a marginal zone. A clear distinction between the red and white pulp was evident in the spleens of normal control rats (Figure 5A) and okra control animals (Figure 5D). Microscopic inspection of spleen sections of DM rats revealed that the white pulp was widely dispersed, and mature lymphocytes in the periphery was also drastically decreased (Figure 5B), with thick follicular arteries, necrosis, and depletion of lymphocytes in the germinal center (Figure 5B). Spleens from diabetic animals treated with insulin showed mildly thickened follicular arteries and mild lymphocytic depletion in the germinal center (Figure 5C). However, spleen sections from diabetic rats treated with okra showed an obvious improvement, with a normally distributed lymphocyte population and a normal ratio relative to red pulp (Figure 5E). According to the statistical examination of histological abnormalities in splenic tissues, diabetes generated significant depletion, degeneration, and necrotic changes in lymphocytes of white pulp. As compared to control animals, treatment with okra markedly restored these parameters to levels that are close to normal (Figure 5F).

**Figure 6:**
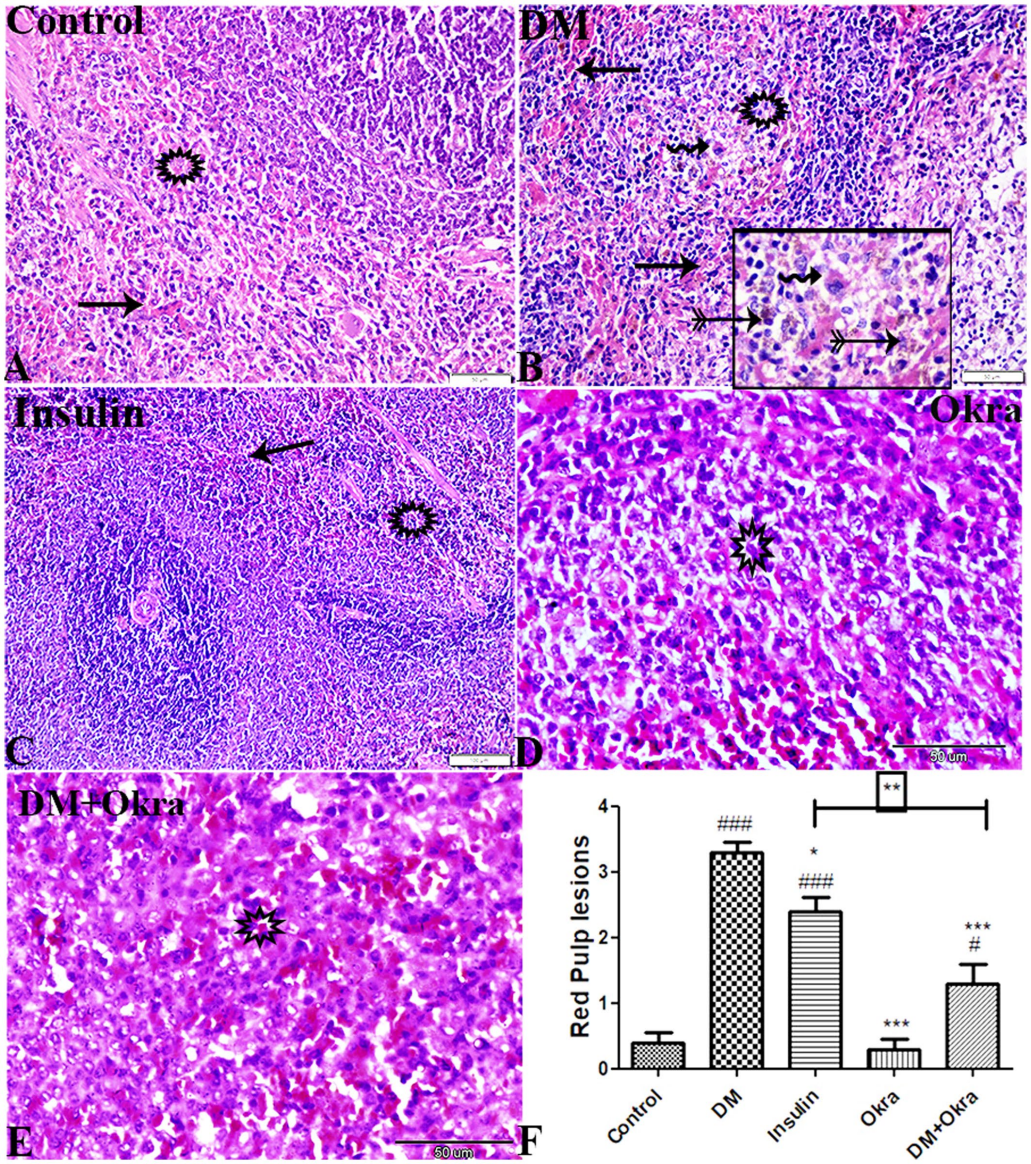
Photomicrograph of spleen tissue sections (Red Pulp) stained with HE stains **(A)** control group: normal red pulp containing normal lymphoid cellular density (star), splenic cords and sinusoids (arrow). **(B)** DM untreated group: congested sinusoids (arrows), necrosis and depletion in lymphocytes (star), **(B)** magnified in selected square: marked sidrophage cell numbers (notched arrows), megakaryocytic cells (zigzag arrows). **(C)** Diabetic group treated with insulin: a normal histological appearance of red pulp composed of condensed lymphocytes (star) splenic cords and sinusoids (arrow). **(D)** Okra control group: **(E)**: DM + Okra, showing improvement in red pulp structure. The bar size represents 50 μm, **(C,D)** represents 100 μm. **(F)** Histomorphometry graph showing semiquantitative measurements of splenic red pulp total lesion scores. Data are expressed as means ± standard deviations. Significant differences versus the control group are marked by #, while significant differences versus DM group are marked by different asterisks through one-way ANOVA with Tukey’s *post hoc* test: ^#,^ *p ≤ 0.05, **p ≤ 0.01, ^###,^ ***p ≤ 0.001.

The histological examination of splenic tissue from the control group and the okra control group revealed that the red pulp contained lymphoid cells, plasma cells, reticular fibers, splenic cords, and sinusoids (Figure 6A). The examination of spleen sections from diabetic rats revealed severe congestion and degenerative changes, deposition of hemosiderin granules (characteristic of damaged spleen) represented by marked siderophage (macrophages that engulf hemosiderin to give yellow-brown granules), and lymphocyte depletion (Figure 6B). Interestingly, the DM + Okra group (Figure 6E) showed partial repair of the red pulp architecture similar to the DM + Insulin group (Figure 6C), and similar to the control rats treated with Okra extract (Figure 6D). When compared to the tissues of control groups, the statistical analysis of the histopathological changes in the splenic tissues showed that diabetes significantly caused various lesions in the red pulp, while Okra treatments significantly restored the histopathological changes to near normal levels (Figure 6F).

### 3.5 Collagen deposition in spleen

**Figure 7:**
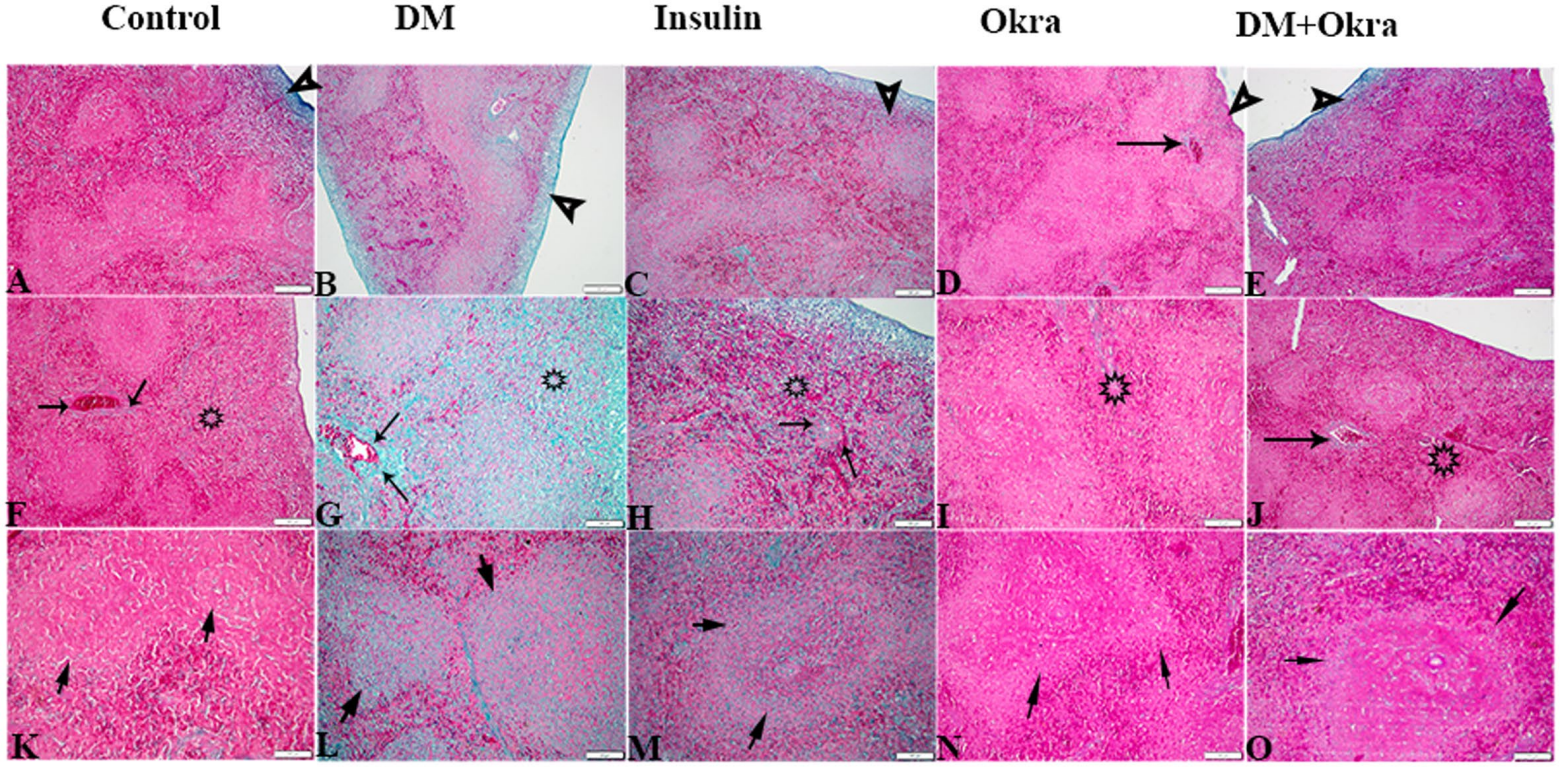
Photomicrograph of spleen tissue sections stained with Masson’s Trichrome from the experimental groups showing the collagen deposition in **(A–E)** the splenic tissue capsule (arrowheads). **(F–J)** Collagen deposition in PALS (arrows), and in red pulp (stars). **(K–O)** Collagen deposition in splenic white pulp (arrows). The bar size represents (**A–F** = 200 μm), (**G–O** = 100 μm).

**Figure 8:**
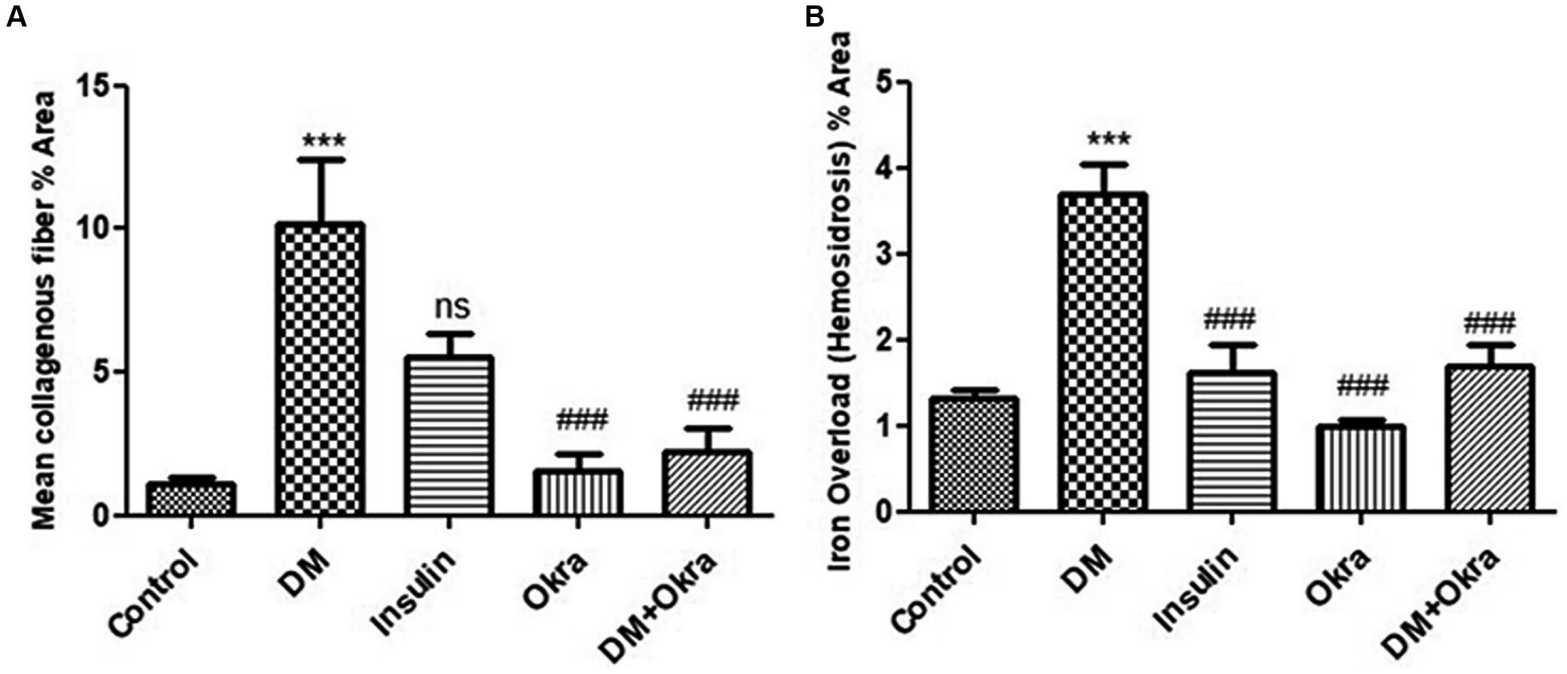
Histomorphometry graph showing quantitative measurements representing mean percentage area of **(A)** Collagenous fibers, **(B)** Iron overload (hemosiderosis) in all groups. Data are expressed as means ± standard deviations. Significant differences versus the control group are marked by #, while significant differences versus DM group are marked by different asterisks through one-way ANOVA with Tukey’s *post hoc* test: (^###,^ ***p ≤ 0.001), ns: non-significant.

Figures 7A,F,K show a representative section of the control spleen, while Figures 7D,I,N show a representative section of the spleen from the okra control group. Additionally, it was noted that the spleens of diabetic rats had increased capsule thickness with widely dispersed trabeculae and a relatively high degree of fibrosis surrounding the follicular artery (PALS), in both the white and red pulps (Figures 7B,G,L). DM rats treated with insulin showed moderate collagen deposition in capsule and red and white pulp (Figures 7C,H,M). In contrast, sections from the diabetic rats received okra displayed normal splenic trabeculae distribution and capsule thickness (Figures 7E,J,O). When compared to the control tissues, okra treatments were observed to dramatically return these parameters to normal levels (Figure 8A).

### 3.6 Iron overload (hemosiderosis) in spleen

**Figure 9:**
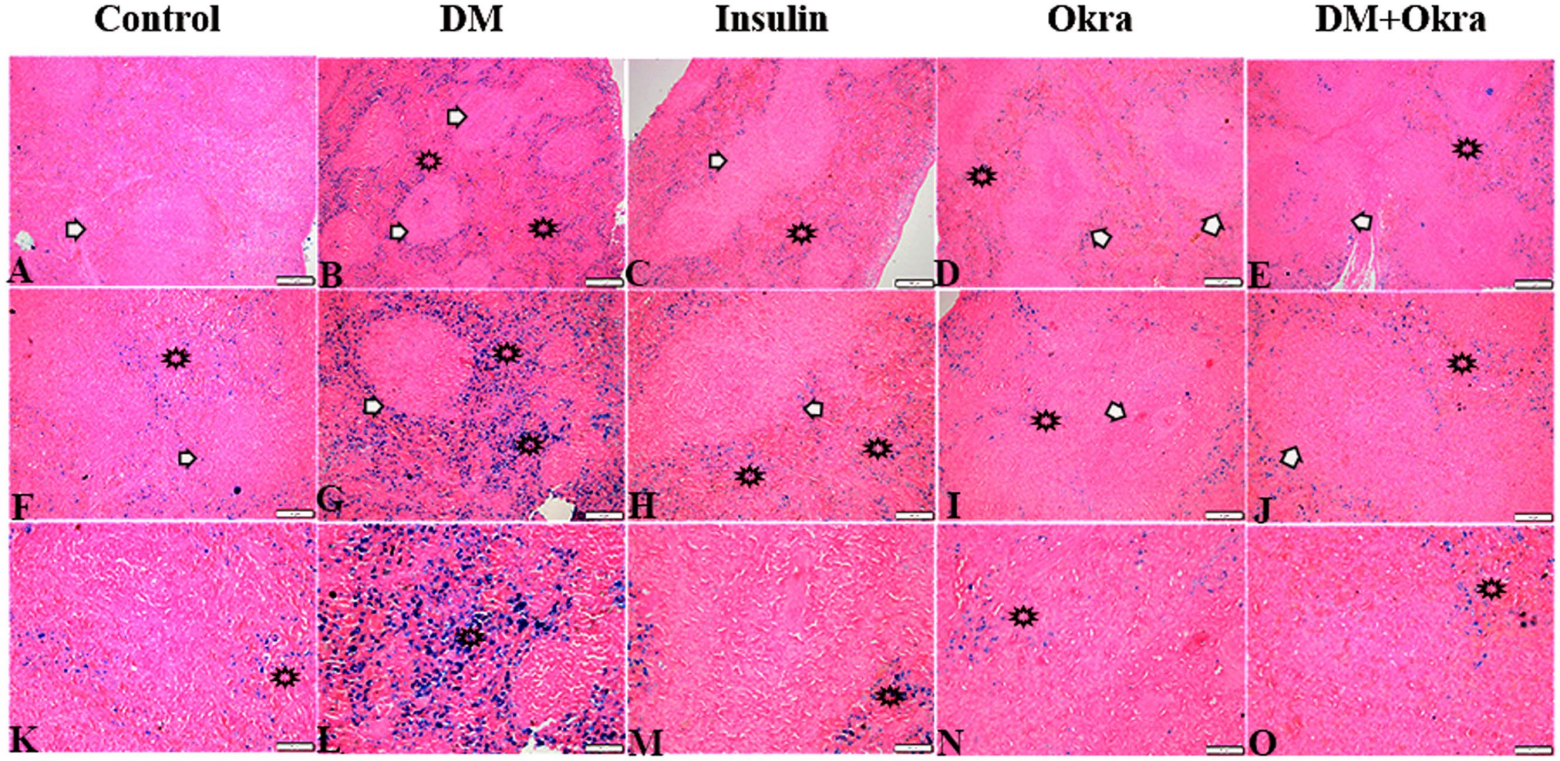
Photomicrograph of spleen tissue sections stained with n stained with Perl’s Prussian bluefrom the experimental groups showing the erythrophagocytic activity and iron overload (hemosidrosis) in the splenic tissue red pulp (stars), and white pulp (arrows). The bar size represents (**A–E** = 200 μm), (**F–J** = 100 μm), (**K–O** = 50 μm).

Histological sections of spleens from the control group (Figures 9A,F,K) and okra control group (Figures 9D,I,N) are shown for comparison. Perl’s Prussian blue staining of sections from the diabetic group revealed diffuse bluish hemosiderosis spots representing precipitations of iron pigment in the white and red pulp of the spleen, indicating marked iron overload (blue granules) (Figures 9B,G,L). Spleens of DM rats treated with insulin showed moderate iron deposition in capsule and red and white pulp (Figures 9C,H,M), but okra treatment was found to restore the histological architecture (Figures 9E,J,O), with blue granule distribution similar to the control group. In comparison to the control tissues, okra treatment significantly restored these parameters to the normal levels (Figure 8B).

### 3.7 Immunohistochemistry assessment

**Figure 10:**
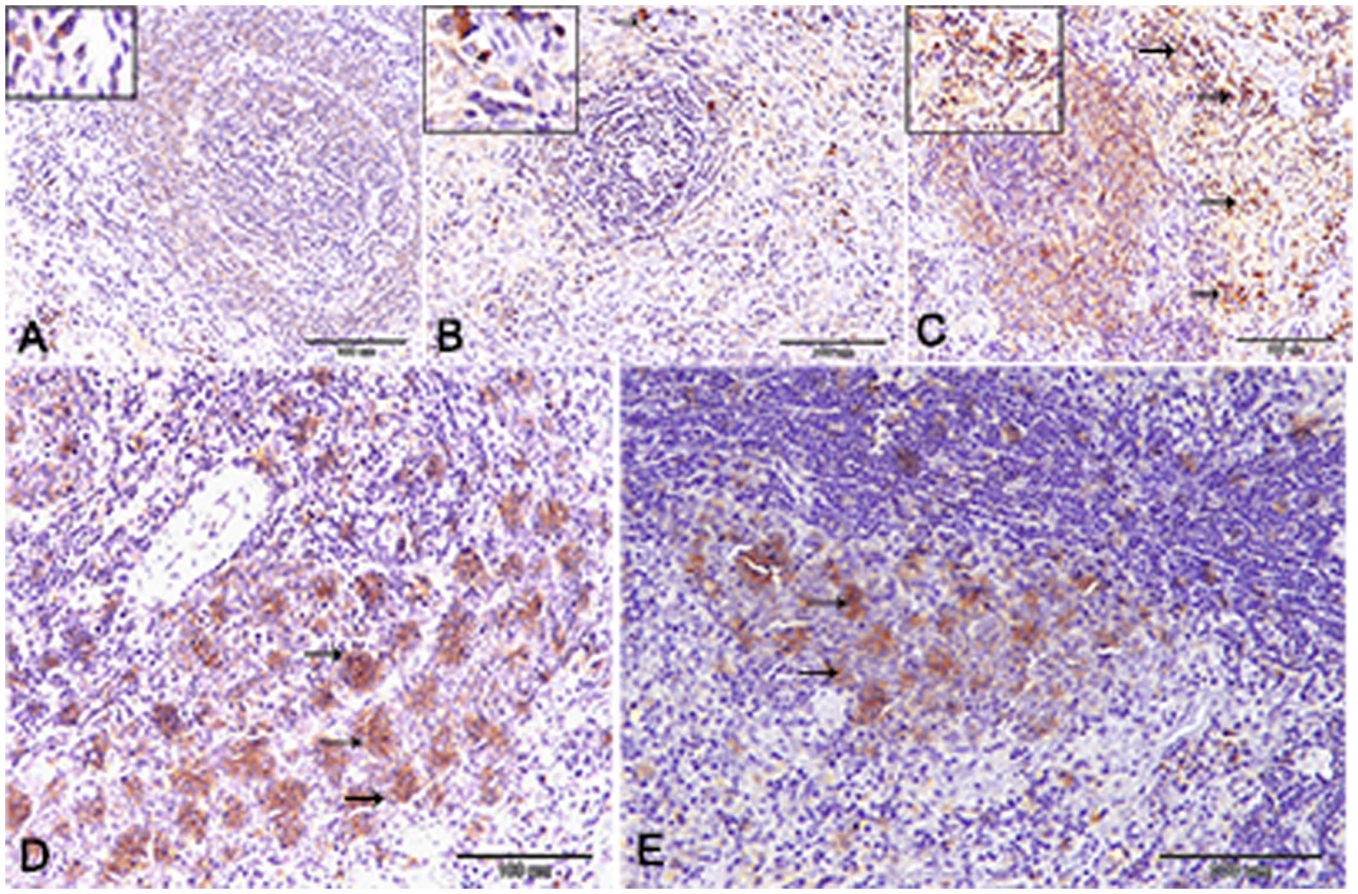
Expression of NF- kβ in the spleen of rats. **(A,B)** Showing only a few numbers of cells in the red pulp of the spleen (inset) from the control and control okra groups reacted positively with NF- kβ. **(C–E)** NF-kβ expression was more pronounced in the red pulp and marginal zone of white pulp (arrows). **(E)** The expression of NF- kβ was mitigated in the diabetic groups that treated with okra.

**Figure 11:**
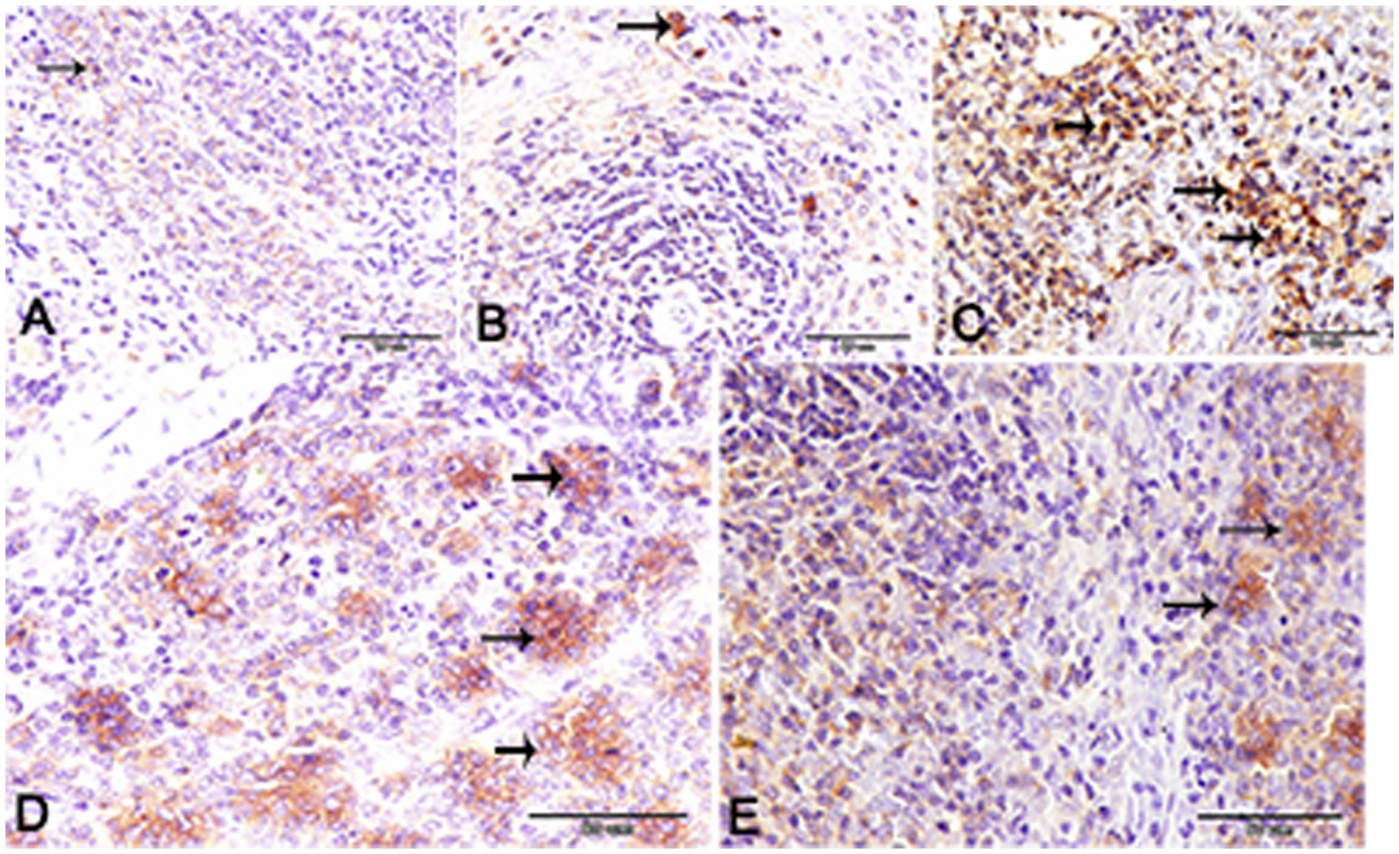
Higher magnification of NF- kβ expression in the different groups. **(A,B)** Showing the control and control okra groups. **(C)** The diabetic group. **(D,E)** The diabetic groups that treated with insulin and okra, respectively.

**Figure 12:**
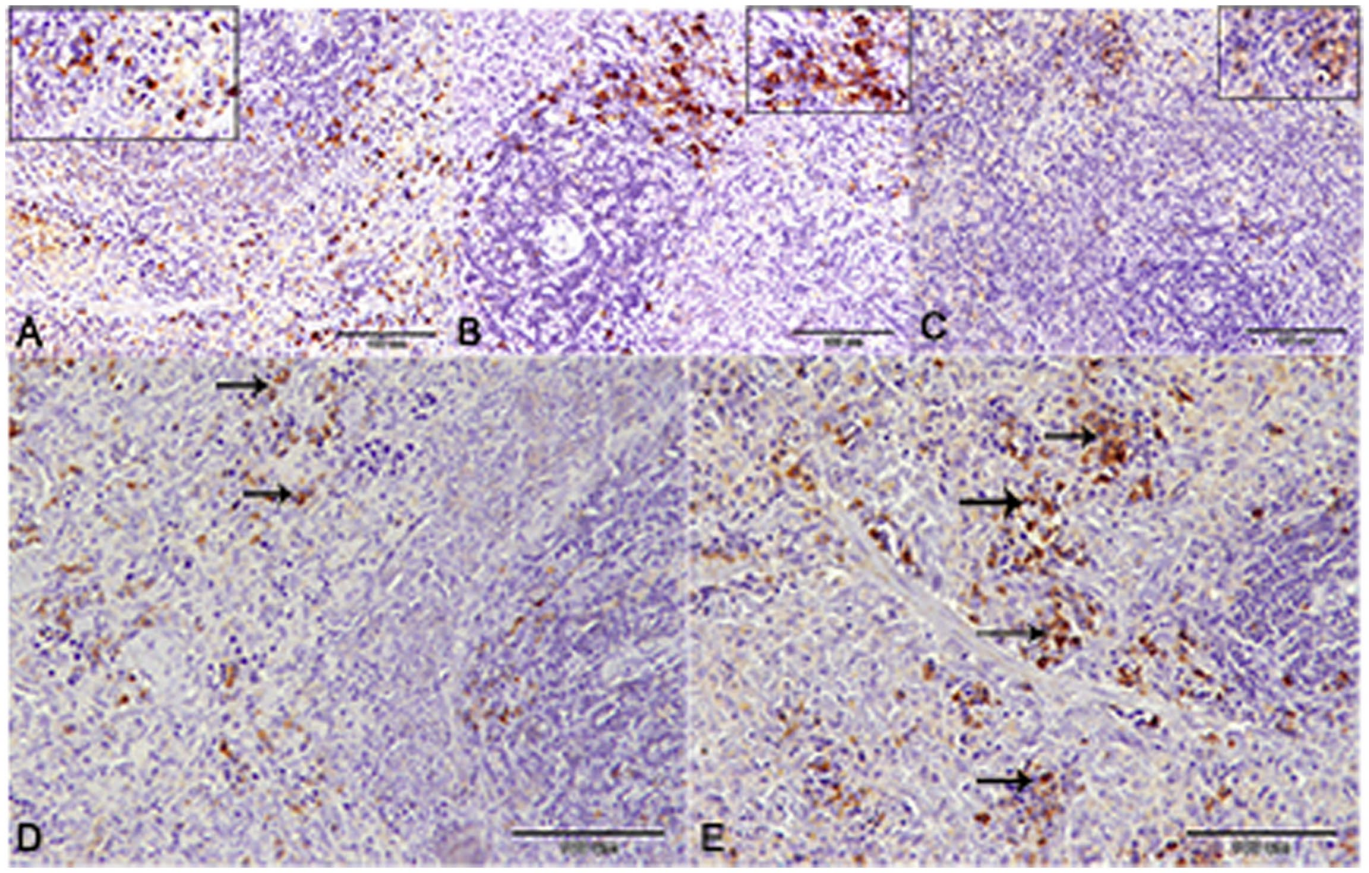
CD-8 expression in the spleen of rats. **(A,B)** Showing the prominent expression of CD8+ T-cells in the control and okra. The expression was more obvious in the red pulp and the marginal zone of the white pulp (inset). **(C,D)** Showing the declined expression of CD8+ T-cells in the diabetic group and the diabetic group that receive insulin treatment. **(E)** The diabetic group that receives okra treatment showed a prominent immunoreactivity to CD8.

**Figure 13:**
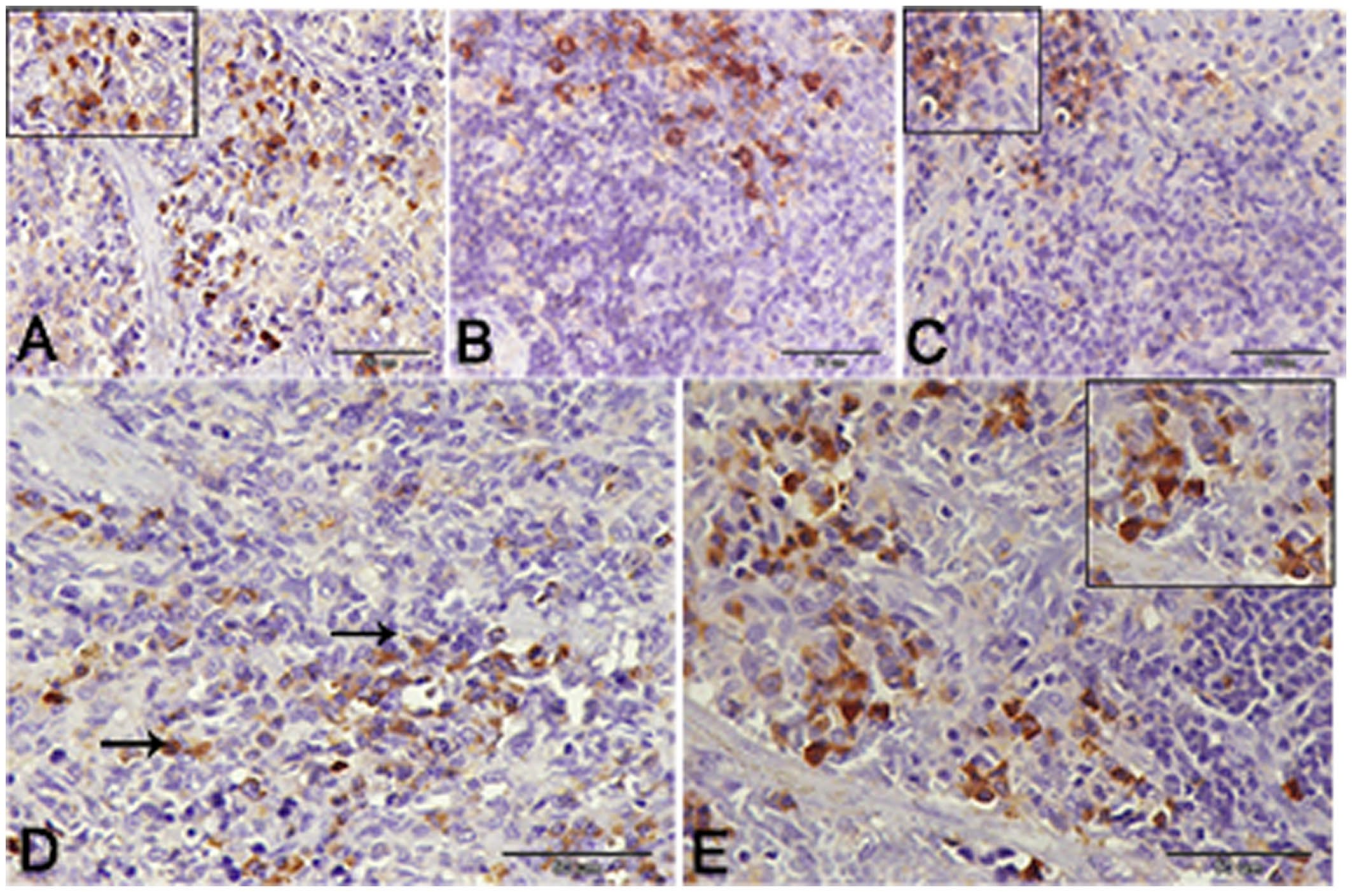
Higher magnification of CD-8 expression in the different groups. **(A,B)** Showing the control and control okra groups. **(C)** The diabetic group. **(D,E)** The diabetic groups that treated with insulin and okra, respectively.

**Figure 14:**
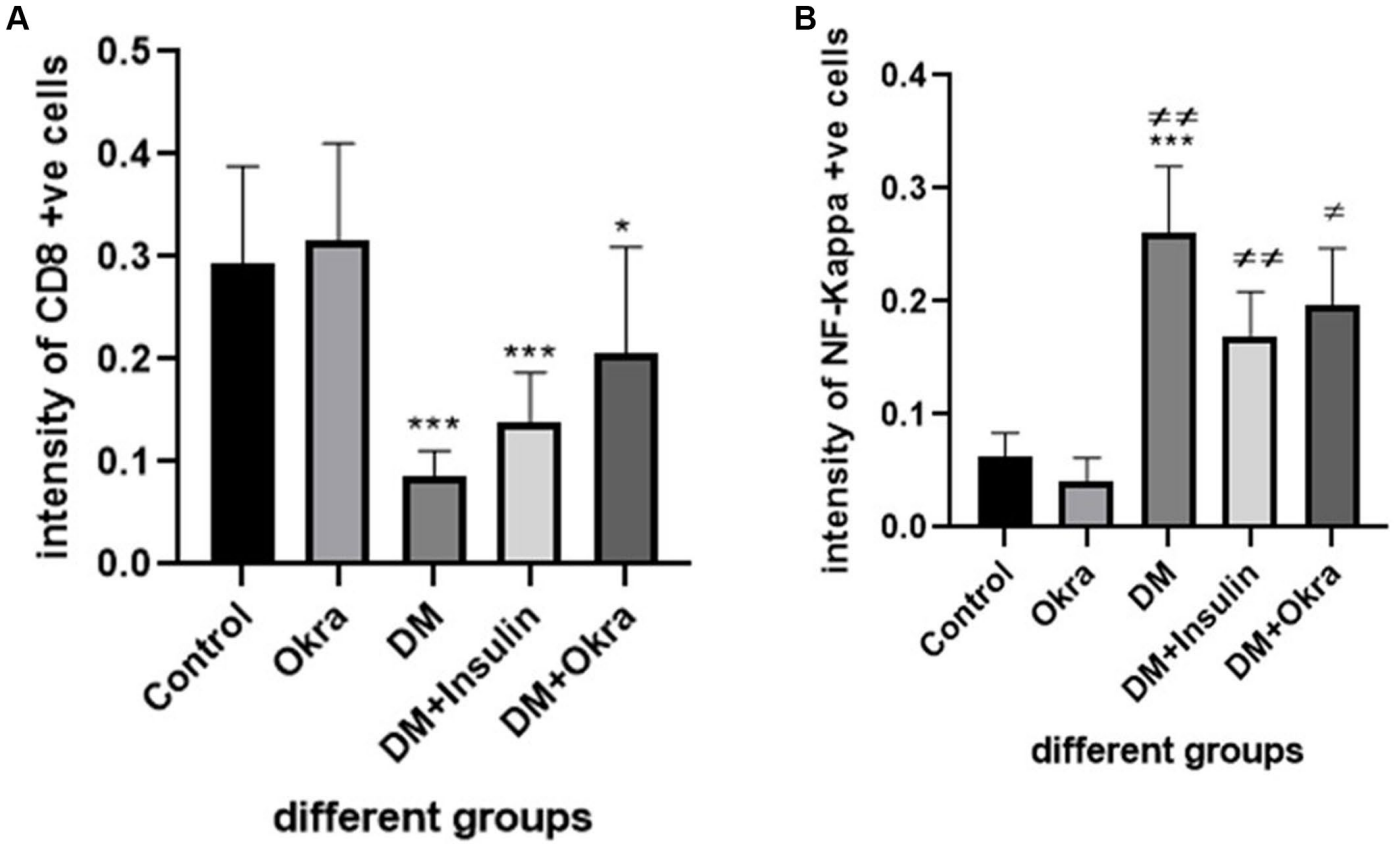
Immunohistochemical staining quantification using image j software. **(A)** Showing the mean immunohistochemistry staining of CD8 in different groups. **(B)** Showing the mean immunohistochemistry staining of NF- kβ expression in the different groups. Data are expressed as means ± standard deviations. Significant differences versus the control group are marked by asterisks. While significant differences versus DM group are marked by different # through one-way ANOVA with Tukey’s *post hoc* test: ***p ≤ 0.001, ^##,^ **p < 0.01, ^#,^ *p < 0.05.

Nuclear factor kappa β (NF-kβ) was investigated with regard to diabetes being implicated as affecting the expression of pro-inflammatory cytokines. Comparing diabetic rat spleens to those of the control and okra control groups, we observed that NF-kβ expression was elevated in the DM rats (Figures 10A–C, 11A–C). However, in diabetic animals that received insulin and okra treatment, the expression was mitigated (Figures 10D,E, 11D,E). In the control and okra groups, NF-kβ expression was restricted to just a few cells in the red pulp of the spleen (Figures 10A,B, 11A,B). However, in the other groups, the expression was more pronounced in red pulp and marginal zone of white pulp (Figures 10C–E, 11C–E). It was found that compared to control and okra control groups, the expression of CD8^+^ T cells were more pronounced in the control and okra groups than in the diabetic groups (Figures 12A–C,13A–C). Comparing the diabetic groups treated with insulin, the immunomodulatory effect of okra on diabetes may be easily clarified (Figures 12D,E, 13D,E). Comparing diabetic groups, DM + Insulin and DM + Okra to control and okra groups, a substantial reduction in the intensity of CD + ve cells was determined (Figure 14A). Comparing diabetic groups to control and okra groups, a substantial increase in the intensity of NF-kβ was determined. NF-kβ intensity was shown to be significantly lower in the DM + Insulin and DM + Okra groups compared to the diabetic groups (Figure 14B).

## 4 Discussion

Type 1 diabetes is characterized by hypoinsulinemia, hyperglycemia, and decreased body weight (41). STZ causes pancreatic β-cell damage and reduces the amount of insulin secreted, which results in hyperglycemia (49). The present work reveals a series of clinical, biochemical, and histopathological findings about the potential benefits of okra in an STZ-induced type 1 diabetic rat model. Similar to several previous studies (50–52), rats with induced DM presented hyperglycemia, glucosuria, and considerable (*p* < 0.05) unexplained weight loss compared with other groups. In our study, okra extract could not restore the weight of rats administered STZ compared with control groups, although compared with the untreated DM group, this body weight result was significant (*p* < 0.05). In the present study, daily administration of 400 mg/kg okra pod extract to DM rats led to reduced fasting blood glucose levels, with values that were close to those of healthy controls. After the first week of the experiment, statistical analysis of fasting blood glucose levels showed that both the diabetic DM and DM + Insulin and DM + Okra groups had considerably (*p* ≤ 0.05) higher levels than the control groups. Before the end of the fourth week, the diabetic group that received okra extract had remarkably lower blood sugar levels (*p* ≤ 0.05) than the untreated DM group. The findings of this study indicate that diabetic rats had significantly high fasting blood glucose levels compared to the control group. Hyperglycemia was dramatically reduced in the groups treated with okra and insulin compared to the diabetic group. According to a previous study, the pancreatic beta-cell membrane is damaged by streptozotocin, and the loss of these cells lowers insulin release (53).

According to the present results, the DM group had the highest level of HbA1c and had a substantially greater level than the control groups (P 0.05). The DM + Okra treated group, on the other hand, had high HbA1c levels that were significantly (*p* ≤ 0.05) higher than those of the control and okra control groups, indicating that the okra treatment had no discernible effect on plasma glucose levels and did not inhibit the development of hyperglycemia in the DM rats. In contrast, the DM group that was not given any treatment, diabetic rats that received okra treatment exhibited a significant reduction in HbA1c levels. This finding agrees with the results of other studies (54, 55). Our findings support a previous study (56) finding that okra may enhance glucose tolerance (57).

In this study, it was found that administering okra pod extract after the onset of diabetes significantly reduced elevated blood glucose levels and led to maintaining body weight, which is consistent with previous reports (58). Importantly, the flavonoids in okra have antioxidant properties that prevent beta cell damage. Additionally, they include oxidative factors that raise insulin levels and can restore beta cells. Okra powder may enhance the glycemic index, according to previous studies (59). Studies have reported that the flavonoids with the highest levels in okra are quercetin and its derivatives (60–62). Flavonoids, including quercetin, have recently been found to lower blood glucose levels (59). According to research by (63), rats given okra exhibited decreased expression levels of the PPARα, PPARγ (Peroxisome proliferator-activated receptors), which were higher in diabetic rats. Nuclear receptor superfamily members PPARα, PPARγ genes have critical functions in modulating cellular proliferation in pancreatic endocrine tissue as well as lipid and glucose homeostasis (64). The polysaccharide extracted from okra has been shown by (33), to have anti-T2DM benefits by lowering oxidative stress through activation of the phosphoinositide 3-kinase (PI3K)/protein kinase B (AKT)/glycogen synthase kinase 3 beta (GSK3) pathway. Additionally, it increased the expression of the nuclear factor erythroid-2 (Nrf2) and promoted the production of the enzyme’s heme oxygenase-1 (HO-1) and superoxide dismutase 2 (SOD2), which are both mediated by Nrf2.

According to the present study, blood glucose levels were significantly lower in the DM + Okra group than in the DM group. A number of previous studies have shown that dietary supplements containing natural antioxidants, such as flavonoids and phenolic compounds, can reduce the risk of streptozotocin-induced diabetes (65). Thus, pancreatic tissue may benefit from the flavonoids and fiber present in okra (65, 66). After 4 weeks, a significant difference was found between the groups. Therefore, blood sugar levels in diabetic rats can be reduced if they consume okra extract for 4 weeks.

Spleens are made up of two distinct elements: a white pulp and a red pulp. T and B lymphocytes, as well as macrophages, make up the majority of the white pulp (56). However, parenchymatous cells and vascular sinuses make up red pulp. A notable histological characteristic of the diabetes control group is a decrease in white pulp and activation of red pulp in the spleen. An early sign of spleen damage is hemosiderin, an iron-loading complex made of ferritin crystals (67).It should be noted that persistent hyperglycemia is the most common cause of multiple organ failure (68). One of the most serious effects of diabetes is destruction of the spleen, a secondary lymphoid organ made up of red and white pulp sandwiched between two portions in the marginal zone (13, 68). A fundamental function of red pulp is blood filtration, by removing damaged erythrocytes (56). White pulp, which contains T- and B-lymphocytes, dendritic cells, and macrophages, is important for the spleen’s immunological function, which protects against infection (56, 69). Antigen processing occurs in the marginal zone (56, 70). Diabetes, according to research, produces morphological and histopathological abnormalities as a defining hallmark of spleen damage. These changes can eventually lead to immunological dysfunction (13, 71, 72). Splenic immune dysfunction in both diabetic rats and humans has been reported in previous studies (13, 73). Splenic malfunction can also disturb glucose and lipid metabolism (13, 73). This might explain the potential influence of the spleen’s metabolic and immunological responses in the development of diabetes (13, 73, 74). In line with this, there is unambiguous proof that splenectomy and hyperglycemia are associated (73).

The depletion of white pulp and its marginal zone was significantly reduced in diabetic rats was previously described (75), while the volume density of the red pulp zone increased, pointing to spleen degradation and a diminished cellular immunological response. Supplementation with okra lessened diabetes’s harmful effects. This was demonstrated by the improvement in size and cellular density of the white pulp and marginal zone, indicating the ability to lessen spleen damage in diabetes conditions.

There is also evidence that okra protects against diabetic-induced splenic damage. Oxidative stress is generated by the overproduction of free radicals and a depleted antioxidant defense, which is recognized as a key regulator in the process of splenic apoptosis in diabetes (52, 68, 75). In the diabetic condition, high glucose concentrations induce ROS release by activating the glucose autoxidation, hexosamine, protein kinase C, and polyol pathways, as well non-enzymatic protein glycation, which together contribute to splenic damage (68, 76). Excess ROS production induces lipid peroxidation, which eventually yields components that can accelerate cell death signaling and trigger cell death (77, 78). ROS overproduction depletes enzymatic antioxidants such as catalase, SOD, and GPx, molecules that function to combat free radicals and neutralize oxidants (77). Taking this into account, splenic cell damage in diabetes is primarily due to the excessive oxidative stress generated by a pro-oxidant/antioxidant imbalance. In light of this finding, increased oxidative stress caused by a pro-oxidant/antioxidant imbalance is the main cause of splenic cell damage in diabetes. In previous studies, it was shown that STZ-induced diabetes in rats was associated with an obvious increase in splenic oxidative stress (13, 52, 68, 75). Concerning the antioxidative effects of flavonoids, okra aided in prevention of oxidative damage by enhancing endogenous antioxidant defense and scavenging free radicals that were produced (52, 79, 80). The antioxidant and free radical scavenging properties in okra pod extract effectively alleviate spleen damage caused by hyperglycemia. Histological sections of spleen have shown that okra treatment after induction of diabetes reverses the reduction of white pulp, activation of red pulp, and augmented hemosiderin deposition, indicating that okra has the potential to restore normal immunological functions of the spleen. Histological analyses of the spleen have demonstrated that treating animals with okra after the onset of diabetes reverses the circumstances of reduced white pulp, activated red pulp, and increased hemosiderin deposition, suggesting that okra has the potential to reestablish the normal immunological functions of the spleen. The pathogenesis of type 1 diabetes has been linked to increased amounts of inflammatory cytokines, which may help attract macrophages and lymphocytes to the sites of inflammation (81).

Nuclear factor kappa β (NF-kβ) is a transcription factor that has been demonstrated to be activated in response to stress or signals produced by pathogens. NF-kβ is quiescent in the cytoplasm of cells and is not activated unless an appropriate type of cellular stimulation occurs (82). NF-kβ controls cell proliferation, adhesion, apoptosis, and angiogenesis in a variety of cell types (83). The inflammatory proteins interleukin 6 (IL6) and tumor necrosis factor alpha (TNF-α) are produced as a result of the NF-kβ activation (84). The pathophysiology of diabetes is significantly influenced by the expression of cytokines and inflammatory substances (85). According to (86), the NF-kβ family of transcription factors consists of five closely related transcription factors: p50 (NF-kb1), p52 (NF-kb2), p65 (RelA), c-Rel, and RelB. the antidiabetic benefits of dietary flavonoids and quercetin and their underlying molecular mechanisms on particular pathways, including the glucose transporter, hepatic enzymes, tyrosine kinase inhibitor, NF-kβ, AMPK (5′ adenosine monophosphate-activated protein kinase) and PPAR. Through the control of glucose metabolism, hepatic enzyme activity, and a lipid profile, flavonoids ameliorate the pathogenesis of diabetes and its consequences (87). Additionally, quercetin’s modulatory effect on NF-kβ, a nuclear factor kappa-light-chain-enhancer of activated B cells, aids in enhancing the release of insulin induced by glucose (88). Comparing the spleens of DM rats to those in the control and okra control groups, we observed that NF-kβ expression was exaggerated in the DM rats. However, in diabetic animals that received insulin and okra treatment, the expression was mitigated. Therefore, we were able to show the ameliorative effect of okra on diabetic animals: compared to diabetic rats, animals treated with okra had decreased positive NF-kβ immunostaining.

Innate and adaptive immunity are regarded as important immune system components. It should be emphasized that the two primary adaptive immunity mediators are B cells, which produce antibodies, and T cells, which are further classified into helper CD4+ cells and cytotoxic CD8+ cells (89). Patients with diabetes mellitus have impaired function of both CD4^+^ and CD8^+^ T cells (90, 91). Therefore, diabetes progression is substantially influenced by abnormal immune cell activation and the subsequent inflammatory environment. In the majority of pancreatic biopsies from T1D patients, class I human leukocyte antigen (HLA) is hyper-expressed in islet and endothelial cells (92). These findings imply that cytotoxic T lymphocytes’ identification of islet autoantigens delivered by class I HLA molecules may play a significant role in the effector mechanism that attacks beta cells (93). The current study demonstrates the immunomodulatory effect of okra on the adaptive immune response in streptozotocin (STZ)-induced diabetic rats. This was evidenced by the expression of CD8^+^ T cells, which was more obvious in diabetic animals treated with okra compared to diabetic animals and diabetic animals treated with insulin. According to previous research (94), diabetic rats have significantly fewer lymphocytes in their spleen and peripheral blood. This finding suggests that elevated levels of free radicals, rising pro-inflammatory cytokine levels, and programmed cell death are all signs of diabetic toxicity stressing lymphocytes.

## 5 Conclusion

Okra pod extract exhibited potent anti-diabetic and anti-hyperlipidemic effects in streptozotocin-induced diabetic rats, indicated by its action in downgrading elevated blood glucose levels and maintaining body weight to a large extent. Okra pod extract effectively alleviated spleen damage caused by hyperglycemia. Histological sections of the spleen demonstrated that okra administration after diabetes induction reversed the reduced white pulp and activated red pulp, and increased hemosiderin deposition, indicating its powerful effect on restoring the normal immunological function of the spleen. Okra therapy also lowered inflammation, which is consistent with the other findings. The splenic tissue of the DM group treated with okra had lower NF-kβ expression and higher CD8 expression.

## Data availability statement

The original contributions presented in the study are included in the article/supplementary material, further inquiries can be directed to the corresponding authors.

## Ethics statement

The animal study was approved by this study and all experimental procedures were performed according to the principles of the Ethics Committee of Taif University, Taif, Saudi Arabia (Approval No. HAO-02-T-105) which are in line with the Declaration of Helsinki. The study was conducted in accordance with the local legislation and institutional requirements.

## Author contributions

MA: Writing – original draft, Data curation, Software. KFA: Funding acquisition, Resources, Writing – original draft, Conceptualization, Visualization, Writing – review & editing. AH: Data curation, Software, Writing – original draft, Funding acquisition, Resources, Validation. FA: Data curation, Software, Writing – original draft, Formal analysis, Investigation, Methodology, Project administration, Supervision, Validation, Visualization, Writing – review & editing. MTH: Formal analysis, Investigation, Methodology, Writing – original draft, Writing – review & editing. ASA: Formal analysis, Investigation, Writing – original draft, Resources, Software, Validation. MAH: Formal analysis, Software, Writing – original draft, Methodology. OA-A: Formal analysis, Software, Writing – original draft, Supervision. NA: Software, Writing – original draft, Validation. AAA: Validation, Writing – original draft, Supervision. AJAA: Writing – original draft, Software, Visualization. KSA: Writing – original draft, Supervision, Validation. KJA: Writing – original draft, Formal analysis, Software. AA: Formal analysis, Software, Writing – original draft, Data curation, Supervision, Validation, Writing – review & editing. EA: Data curation, Writing – original draft. NE: Data curation, Writing – original draft, Formal analysis, Investigation, Validation, Visualization, Software. EE: Data curation, Formal analysis, Investigation, Validation, Visualization, Writing – original draft, Conceptualization, Funding acquisition, Methodology, Project administration, Resources, Supervision, Writing – review & editing.
